# Effect of Epigallocatechin-3-Gallate on EGFR Signaling and Migration in Non-Small Cell Lung Cancer

**DOI:** 10.3390/ijms222111833

**Published:** 2021-10-31

**Authors:** Cristina Minnelli, Laura Cianfruglia, Emiliano Laudadio, Giovanna Mobbili, Roberta Galeazzi, Tatiana Armeni

**Affiliations:** 1 Department of Life and Environmental Sciences, Marche Polytechnic University, 60131 Ancona, Italy; g.mobbili@univpm.it (G.M.); r.galeazzi@univpm.it (R.G.); 2 Department of Clinical Sciences, Marche Polytechnic University, 60131 Ancona, Italy; l.cianfruglia@univpm.it; 3 Department of Science and Engineering of Matter, Environment and Urban Planning, Marche Polytechnic University, 60131 Ancona, Italy; e.laudadio@staff.univpm.it

**Keywords:** epigallocatechin-3-gallate (EGCG), epidermal growth factor receptor (EGFR), tyrosine kinase inhibitors (TKIs), non-small cell lung cancer (NSCLC)

## Abstract

The epidermal growth factor receptor (EGFR) is one of the most well-studied molecular targets in non-small cell lung cancer (NSCLC) and tyrosine kinase inhibitors have been shown to be effective in the treatment of advanced NSCLC. Nevertheless, the efficacy of tyrosine kinase inhibitors could be compromised by additional mutations in EGFR and compensatory activations of other pathways. Epigallocatechin-3-gallate (EGCG), the main bioactive molecule in green tea, acts as a tyrosine kinase inhibitor toward cancer cells overexpressing EGFR (wild-type). However, little information has been reported on the effect of EGCG on EGFR with activating mutations. In this study, we evaluated the ability of EGCG to inhibit EGFR signaling activation in three different NSCLC cell lines containing wild-type EGFR or EGFR with additional mutations. The effect on proliferation, apoptosis, migration, and vinculin expression was then studied. Overall, our results demonstrate that EGCG polyphenol inhibits cell proliferation and migration in NSCLC cell lines, although with different efficacy and mechanisms. These data may be of interest for an evaluation of the use of EGCG as an adjunct to NSCLC therapies.

## 1. Introduction

**Figure 1:**
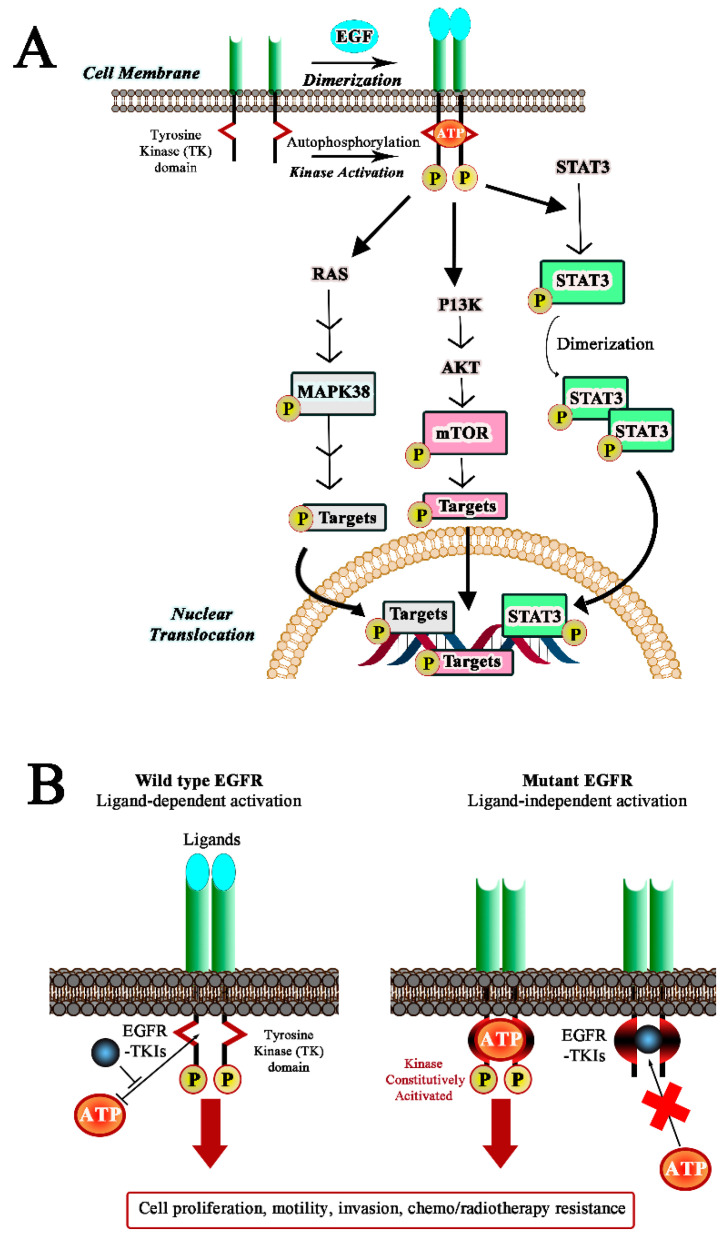
(**A**) Epidermal growth factor receptor (EGFR) pathway. The figure emphasizes the main signaling cascades: mitogen-activated protein kinase (MAPK), signal transducer and activator of transcription 3 (STAT3), and mammalian target of rapamycin (mTOR). (**B**) Differences between the activation mechanism of wild-type and mutant EGFR; the figure shows the role of tyrosine kinase inhibitors (TKIs) in the inhibition of EGFR by competing with ATP for the binding to the tyrosine domain kinase (TK).

Lung cancer is the leading cause of cancer-related death in the world, and non-small-cell lung cancer (NSCLC) accounts for 85% of cases, with most patients generally presenting, at the moment of diagnosis, an advanced lung cancer [1]. The highly invasive and metastatic potential can explain the high mortality rate (25% of all cancer deaths). Many studies have shown that 90% of the NSCLC cases are characterized by overexpression and/or aberrant activation of epidermal growth factor receptor (EGFR) [2,3] which is a cell surface receptor tyrosine kinase to which growth factors selectively bind. Upon ligand binding, EGFR undergoes homo- or heterodimerization with other EGFR family receptors. This leads to the autophosphorylation of its tyrosine residues which serve as specific recruitment sites for downstream signaling molecules including mitogen-activated protein kinase (MAPK), a signal transducer and activator of transcription 3 (STAT3), and mammalian target of rapamycin (mTOR) (Figure 1A). As a result, EGFR, once triggered, acts as the initiator of the signal transduction process, stimulating cell growth and cell migration [4,5,6,7]. As a fact, EGFR overexpression, and/or mutations within the tyrosine kinase domain, lead to an overactivation of the downstream signaling pathways, playing an important role in epithelial cell transformation (Figure 1B).

NSCLC may have overexpressed wild-type or mutated EGFR form. Among the most common EGFR mutations in NSCLC are the in-frame deletion of exon 19 (EGFR^Del19^) and the point mutation in exon 21 (EGFR^L858R^) [8]. EGFR-tyrosine kinase inhibitors (EGFR-TKIs) such as gefitinib (Iressa^®^) [9] have proven to be effective in the treatment of patients with advanced NSCLC harboring these EGFR mutations. Unfortunately, acquired resistance frequently occurs during the treatment. The 50–60% of resistant cases are caused by a secondary mutation on the gatekeeper residue of the ATP-binding pocket of EGFR (T790M, exon 20) [10] which seems to be more inclined to coexist with EGFR^L858R^ than with EGFR^Del19^ [11].

Epigallocatechin-3-gallate (EGCG), is a bioactive catechin present in large quantities in green tea, which shows antiproliferative, anti-inflammatory, and antimutagenic properties [12,13,14,15]. In vitro research demonstrated that EGCG can inhibit the proliferation of lung cancer cells by influencing multiple signal transduction pathways [15,16]. Moreover, its ability to inhibit EGFR phosphorylation in lung cancer cells overexpressing wild-type EGFR (A549 cell lines) is well established [16]. Moreover, the co-administration of EGCG and Gefitinib synergically suppresses the tumor growth in the A549 xenograft mouse model [17].

**Figure 2:**
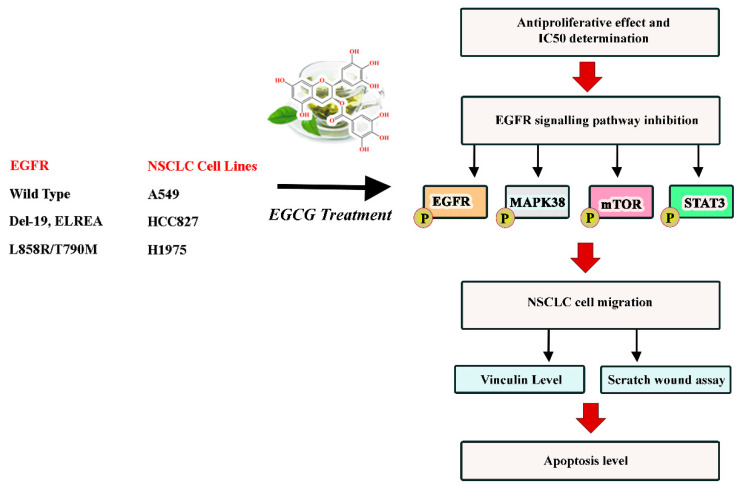
Schematic representation of the study.

However, little information has been reported on the effect of EGCG on the proliferation and migration of NSCLC harboring the in-frame deletion of exon 19 (EGFR^Del19^) and NSCLC with the secondary gatekeeper mutation T790M with oncogene driver L858R (EGFR^L858R/T790M^) [18]. In this study, we evaluate the efficacy of EGCG to inhibit the EGFR signaling pathway by studying the phosphorylation status of EGFR, mTOR, STAT3, and p38-MAPK in NSCLC harboring wild-type EGFR (A549 cell line), in-frame deletion of exon 19 mutation (HCC827 cell line), and L858R/T790M double mutation (H1975 cell line). In addition, the effects of EGCG on cell proliferation, migration, and apoptosis were also evaluated. Since migration and invasion of NSCLC are closely linked to changes in cytoskeleton organization, the effect of EGCG on the expression level of vinculin, the main constituent of focal adhesion, was also determined (Figure 2).

## 2. Results

### 2.1. Effect of EGCG on Viability of Different NSCLC Cell Lines

**Figure 3:**
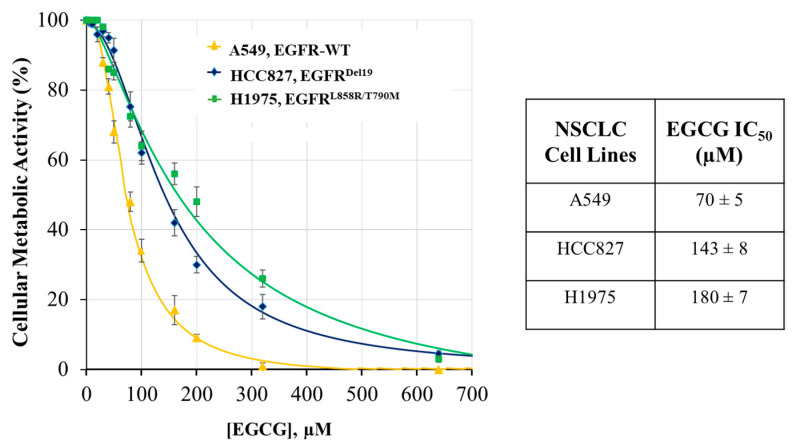
Effect of EGCG treatment on metabolic activity in NSCLC cells. Cell metabolic activity was determined by an MTT assay after exposure to an increasing concentration of EGCG for 72 h. Cytotoxicity curves represent 5 experiments with 5 replicates for each EGCG concentration. Relative IC50 values shown were determined by non-linear regression using the SigmaPlot Software.

The effect of EGCG on cellular metabolic activity (by MTT assay) was evaluated after 72 h of treatment with increasing EGCG concentrations. As shown in Figure 3, although the EGCG dose-dependently decreases the viability of all NSCLC cells, their sensitivity to EGCG treatment is different. A549 cells, with a wild-type EGFR sequence, show an IC50 of 70 µM (SD ± 5). The other two cell lines are more resistant to EGCG treatment. In particular, the HCC827 cells, which are characterized by an in-frame deletion in EGFR exon 19 (EGFR^Del19^), show a two-fold increase in IC50 value with respect to the A549 cells (IC50, 143 ± 8 µM). The H1975 cells, carrying a double EGFR mutation, display a higher drug resistance with an IC50 of 180 µM (SD ± 7). Based on these results, we have chosen values near to ½ IC50 as the three working EGCG concentrations (30, 70, and 90 µM) for the following experiments.

### 2.2. EGCG Differentially Inhibits the EGFR Signaling Pathway

**Figure 4:**
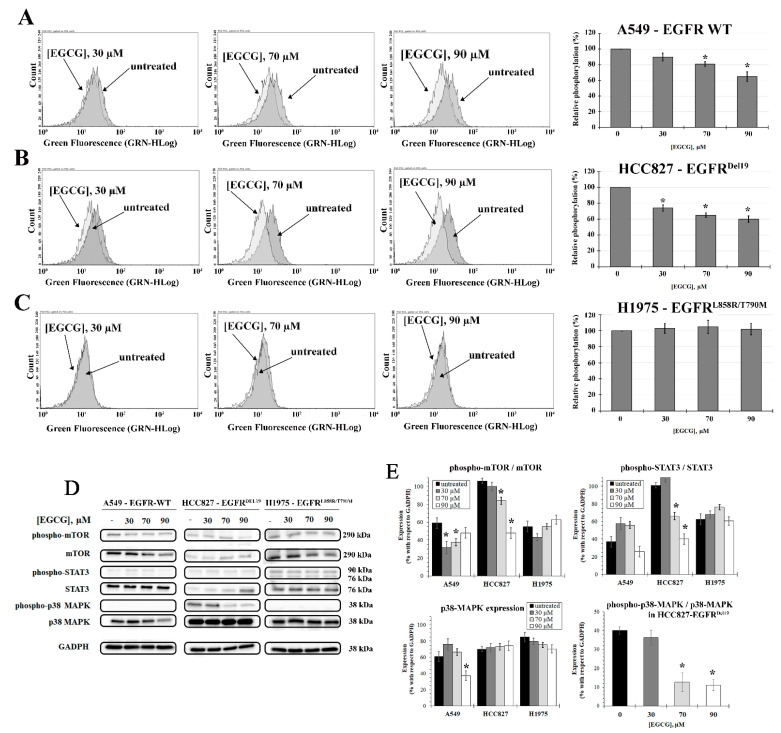
The effect of EGCG on the EGFR signaling pathway. (**A**) A549, (**B**) HCC827, and (**C**) H1975 cells after different EGCG treatments (30, 70, and 90 µM) for 24 h and analyzed by flow cytometry after staining with antibodies against EGFR and phospho-EGFR (Y1068). On the left, is shown representative FACS (fluorescence-activated single-cell sorting overlay plots) depicting phospho-EGFR levels after EGCG treatment. The histograms on the right represent the percentages of Y1068 EGFR phosphorylation normalized to total EGFR levels. The data was calculated by dividing the geometric mean fluorescence intensity of phopho-EGFR by the geometric mean fluorescence intensity of the total EGFR. (**D**) Immunoblot analysis for A549, HCC827, and H1975 cells treated with different EGCG concentrations (30, 70, and 90 µM). Cells were lysed 24 h after exposure to EGCG, then extracts were analyzed by western blot with anti-STAT3, anti-phospho-STAT3, anti-mTOR, anti-phospho-mTOR, anti-p38 MAPK, and anti-phospho-p38 MAPK antibodies. GAPDH was used as an internal loading control. (**E**) Densitometric analysis of the ratio between phospho-mTOR/mTOR and phospho-STAT3/STAT3; p38-MAPK expression and phospho-p38-MAPK/p38-MAPK ratio in HCC827 cells. All results are normalized to GAPDH. * *p* < 0.05. For p38-MAPK and phospho-p38-MAPK, a longer exposure time was used (15 s). Full-length blots are presented in Supplementary Figure S1. The samples were derived from the same experiment and the blots were processed in parallel.

In order to investigate how the EGFR mutations affect the ability of EGCG to inhibit the activation of EGFR signaling pathways, phosphorylation studies on EGFR, mTOR, STAT3, and MAPK38 have been performed (Figure 4).

As shown in Figure 4A–C, the mutational status of EGFR directly influences the binding of EGCG, resulting in a different efficacy of the molecule in the inhibition of EGFR phosphorylation. In the A549 cell line, EGCG induces a dose-dependent decrease of EGFR phosphorylation reaching, at 90 µM, an inhibition of about 40% with respect to untreated cells (*p* < 0.05). The HCC827 cells show the highest sensitivity with a 20% decrease in EGFR phosphorylation already at 30 μM EGCG which significantly increases in the presence of 70 and 90 μM EGCG (40% decrease with respect to untreated cells, *p* < 0.05). No effect of EGCG on EGFR inhibition has been observed in the H1975 cell line.

To determine the role of mTOR in EGFR signaling, western blotting of mTOR and phospho-mTOR has been performed. As shown in Figure 4C,D, ECGC induces a significant decrease in the phosho-mTOR/mTOR ratio in the HCC827 cell line (1.6- and 3.5-fold decrease of phospho-mTOR/mTOR ratio at 70 and 90 µM EGCG concentrations, respectively) (*p* < 0.05). In A549 cells, 30 and 70 μM EGCG induce a significant reduction in the phospho-mTOR/mTOR ratio (twofold decrease with respect to untreated cells, *p* < 0.05). Although EGCG induces a decrease in mTOR expression and mTOR phosphorylation, the phospho-mTOR/mTOR ratio remains unchanged in H1975 cells (Figure 4D,E).

The inhibition of STAT3 signaling was observed only for the HCC827 cell line in which there is a decrease in phospho-STAT3/STAT3 ratio, at 70 and 90 µM EGCG concentration, of about three times with respect to untreated HCC827 cells (*p* < 0.05). No effect is present for the other two NSCLC cell lines.

Expression of p38 MAPK is affected by EGCG treatment only in the A549 cells at 90 μM EGCG (twofold decrease with respect to untreated cells, *p* < 0.05); the phosphorylated form of p38 MAPK (phospho-p38 MAPK) is present only in the HCC827 cell line (Figure 4D). In fact, as just described, phospho-p38MAPK is usually poorly detectable in cells [19]. HCC827 cells did not show any significant changes in the p38 MAPK expression but a strong reduction in its phosphorylated form is observed after 24 h of treatment with 70 and 90 µM EGCG concentrations (fourfold decrease with respect to untreated cells, *p* < 0.05).

### 2.3. EGCG Differentially Affects Cellular Migration and Vinculin Expression

**Figure 5:**
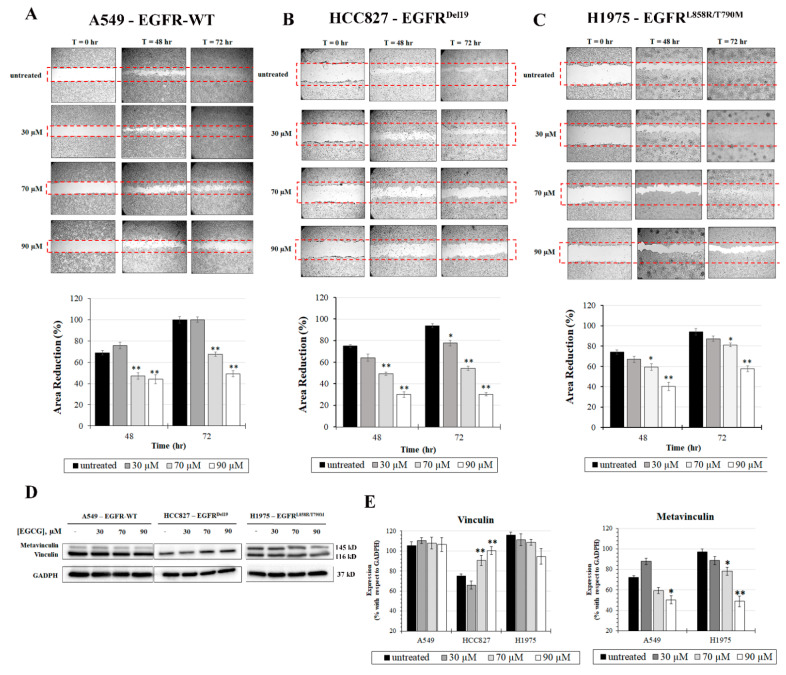
The effect of EGCG on NSCLC cell migration. (**A**) A549, (**B**) HCC827, and (**C**) H1975 cell migration after different EGCG treatments (30, 70, and 90 µM) was evaluated by scratch wound assay. Images were taken at times 0, 48, and 72 h. The wound area was measured by ImageJ software. The percentage migration was calculated by the average area reduction at 24, 48, and 72 h as compared to time 0. The red dotted box represents the size of the original wound. The data is the mean of triplicate experiments ± SD. Scale bar: 100 μm. (**D**) Representative Western blot experiment depicting total vinculin and metavinculin expression after 24 h of EGCG treatment. (**E**) Densitometric analysis showed differences in vinculin expression. GAPDH was used as an internal loading control. * *p* < 0.05; ** *p* < 0.01. GAPDH is the same as Figure 4D as the blots were derived from the same membrane incubated with different antibodies. Full-length blots are presented in Supplementary Figure S1. The samples were derived from the same experiment and the blots were processed in parallel.

To understand the effect of EGCG on NSCLC cell migration, an in vitro scratch model was used, and the results were expressed as a percentage of wound closure. Treatments have been evaluated after 48 and 72 h in the presence of the selected EGCG concentrations (Figure 5).

After 72 h of 30 µM EGCG treatment, the migratory ability of A549 cells is not affected while, at 70 µM EGCG, it is significantly retarded by 33% when compared to untreated cells (*p* < 0.05). The inhibition effect further increases in the presence of 90 µM concentration where the wound closure decreases by 52% after 72 h of treatment (*p* < 0.05). In the H1975 cell line, EGCG has a dose-dependent effect on cellular migration. In particular, after 72 h of treatment with 70 and 90 µM EGCG, the percentage of area decreases by 20 and 40%, with respect to reference control value, respectively (*p* < 0.05). In contrast, EGCG strongly affects the migratory ability of HCC827 cells, with a marked dose-dependent effect. After 72 h of treatment, EGCG 30 µM induces an inhibition of 20% in the migration rate while, at 70 µM, EGCG retards the wound closure by 45%. Interestingly, at 90 µM, the cell migration rate is completely arrested and the wound area is the same at 48 and 72 h of treatment while only 30% of cells migrated in the wound with respect to untreated cells (Figure 4).

Since the speed of wound closure depends, in addition to cell proliferation index, on cytoskeleton reorganization, the impact of EGCG on the expression of vinculin (Vnc) was studied by immunoblot results (Figure 5D,E). The anti-vinculin antibody detects two isoforms of Vnc in A549 and H1975 cell lines, vinculin (116 kDa) and metavinculin (145 kDa), resulting in two bands on the immunoblot. Although no effect is observed in the Vnc expression after EGCG treatment, a reduction in metavinculin (mVnc) expression is observed. In particular, at 90 μM, EGCG causes a significant decrease in mVnc expression of about twice in H1975 cells with respect to untreated cells (*p* < 0.01). A similar effect is observed in the A549 cells with a 1.5-fold decrease in mVnc expression (*p* < 0.05). Interestingly, the mVnc isoform is not revealed in HCC827. Furthermore, in this case, EGCG significantly increases the Vnc expression level already at 70 µM of about twice with respect to control (*p* < 0.01).

### 2.4. EGCG Treatment Induces an Increase in NSCLC Apoptosis Level

**Figure 6:**
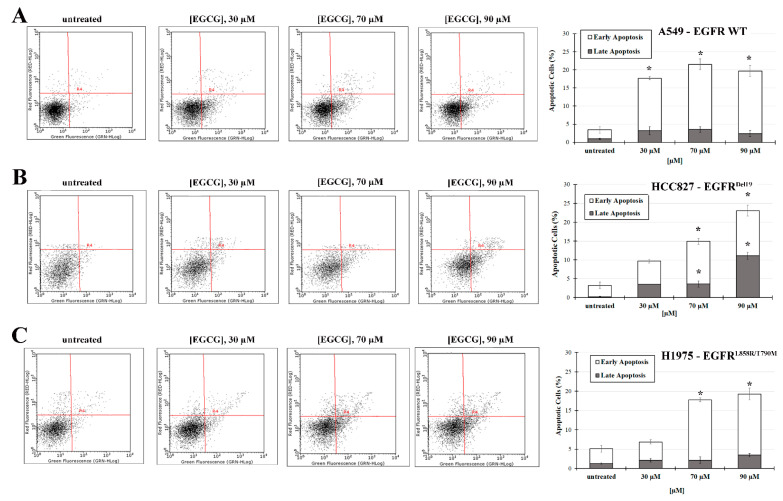
The effect of EGCG on NSCLC apoptosis level. Flow cytometry analysis of (**A**) A549, (**B**) HCC827, and (**C**) H1975 cells after 72 h with different EGCG treatments (30, 70, and 90 µM) using Annexin V/FITC and PI double stain. GRN-HLog refers to the green fluorescence intensity (FITC channel) and RED-HLog to the red fluorescence intensity (PI channel). On the left there are representative cytograms in which R4 indicates the markers used for the analysis to identify the state of the cells: viable (Annexin V-PI-), early apoptotic (Annexin V+PI-), late apoptotic (Annexin V+PI+), and necrotic (Annexin V-PI+) cells. The histograms on the right represent the percentages of early and late apoptosis. The data is the mean of triplicated experiments ± standard deviation. * *p* < 0.05.

To detect apoptosis, Annexin V-FITC, a calcium-dependent phosphatidylserine-binding protein, has been used. During both early and late apoptosis, apoptotic cells or bodies translocate phosphatidylserine to the outer leaflet of the plasma membrane; therefore, fluorescent Annexin V conjugate can detect cells at different apoptotic stages. Figure 6 shows the apoptosis levels of the three NSCLC cell lines untreated or treated with the different concentrations of EGCG after 72 h.

Untreated A549 cell lines show 95% live cells with an intact physical shape whereas the cells treated with EGCG show significantly increased cell death rates already at 30 µM with 14% of early apoptotic cells and 3% of late apoptotic cells. The concentrations of 70 and 90 µM cause an even greater increase in the percentage of early apoptotic cells to 18%.

HCC827 cells show a dose-dependent increase in apoptosis levels. Indeed, at 30 µM EGCG, there is an increase in the percentage of apoptotic cells, from 3% of untreated cells to 10%. At 70 µM of EGCG concentration, the percentage of cells undergoing apoptosis reaches 15%, of which 11% undergo early apoptosis and 4% late apoptosis. The 90 µM concentration of EGCG further increases the percentage of cells in apoptosis with respect to untreated cells (23%); a significant increase in late apoptosis is observed.

H1975 cells are also sensitive to the treatment with 90 μM EGCG, which significantly increases the rate of apoptosis to 19% mainly by early apoptosis induction.

## 3. Discussion

Concerning the EGFR overexpression or mutation, there is a constitutive activation of EGFR downstream signaling pathways, which include PI3K/AKT/mTOR pro-survival, STAT3 transcription factor, and RAS/MAPK proliferation pathways [20,21]. Once activated, this complex network of cellular signal transducers induces cell proliferation, survival, and cell migration. Therefore, in NSCLC stimulation of proliferation and survival is induced by mutated EGFR, which is constitutively active. Since its function is finely regulated by reversible protein phosphorylation, tyrosine kinase inhibitors (TKIs) are widely used in the treatment of NSCLC. However, although some studies have shown very high response rates of EGFR-mutant tumors to TKIs, direct and/or indirect resistance mechanisms could interfere with the efficacy of the latest TKIs. Known resistance mechanisms involve a secondary mutation (T790M) and a tertiary mutation (C797S) in patients treated with gefitinib and osimertinib, respectively. In addition, compensatory activations of downstream EGFR pathways, such as MAPK, STAT3, and mTOR, could bypass the EGFR signaling inhibition [22,23]. It is therefore an urgent need to discover molecules able to bypass these resistance mechanisms reinforcing the efficacy of TKIs. We studied the effect of EGCG on proliferation, apoptosis, and EGFR signaling (mTOR, MAPK38 and STAT3) in three NSCLC cell lines harboring an in-frame deletion of exon 19 (EGFR^Del19^), double mutations T790M/L858R (EGFR^L858R/T790M^), and wild-type EGFR. Since cell motility has been shown to be an integral process in early lung cancer ontogeny, we studied the ability of EGCG to prevent NSCLC migration with respect to the expression of vinculin (Vnc) [24,25].Vnc is a well-known actin-binding protein involved in focal adhesion development acting as a tumor suppressor protein in cancer including in NSCLC [26]. Low Vnc expression is associated with an increased metastatic potential [26,27,28,29].

Our findings show a different sensitivity of NSCLC proliferation toward EGCG treatment (A549 > HCC827 > H1975) (Figure 3) which could be attributed in part to the different ability of EGCG to inhibit EGFR signaling. In wild-type EGFR cells (A549) and in those harboring exon 19 deletion (HCC827), EGCG efficiently inhibits the TK phosphorylation. The results obtained confirm our previous in silico data in which we showed the molecular mechanism of kinase inhibition by EGCG in wild-type EGFR, EGFR^Del19^, and in double mutant EGFR^L858R/T790M^ cells [30]. After the EGFR structure modeling, the estimation of the free-binding energy of EGCG to EGFR-tyrosine kinase domain had been extrapolated using the molecular mechanics with the Poisson–Boltzmann surface area solvation (MM/PBSA) method [31]. The presence of L858R and T790M mutations partially hinders the EGCG binding inside the ATP-binding pocket and therefore the EGCG affinity toward EGFR^L858R/T790M^ drastically decreases (E_MM/PBSA_—54 kcal/mol; K_i_ = 134.11 μM) with respect to the wild-type EGFR (EMM/PBSA —98 kcal/mol; Ki = 0.13 μM). Deletion of the five amino acids in the ATP-binding pocket (EGFR^del19^) leads instead to the formation of additional polar interactions increasing the EGCG binding affinity with respect to EGFR^L858R/T790M^ (EMM/PBSA—75 kcal/mol; Ki = 0.84 μM) [30].

The difference is also found in the phosphorylation of downstream EGFR proteins (Figure 4D,E). In H1975 cells, where the EGFR phosphorylation status is not inhibited by EGCG, the ratio between the phosphorylated form of STAT3 and mTOR and their corresponding total protein does not change with respect to untreated cells. The EGFR signaling pathway is therefore in an active state in presence of EGCG. These results indicate that the treatment of EGFR^L858R/T790M^ positive NSCLC patients would not strengthen the TKIs efficacy. However, at 70 and 90 µM of EGCG, a significant induction of apoptosis is observed in the H1975 cell line (Figure 6), probably due to the inhibition of other pathways. EGCG is in fact a pleiotropic molecule able to interact with several biological targets. Moreover, after 72 h of treatment, EGCG (90 μM) strongly reduces the migration of H1975 cells with an inhibition of wound closure of about 40% with respect to untreated cells, and this could be related to a decrease in the expression of meta-vinculin (mVnc) (Figure 5). This is a Vnc splice isoform that contains an additional exon encoding a 68-residue insert within the actin-binding tail domain. Similarly to Vnc, mVnc can engage filamentous (F)-actin, but only vinculin can promote F-actin bundling [32,33,34]. Moreover, several studies have shown that mVnc can negatively regulate Vcn-mediated F-actin bundling in vitro. This novel finding could explain the EGCG-induced decrease in cellular migration in addition to the apoptosis induction. Overall, EGCG suppresses cell proliferation independently of EGFR signaling pathway inhibition in the H1975 cell line while inducing a significant decrease in cell migration.

As just reported [35], the inhibition of EGFR phosphorylation in A549 cells, overexpressing wild-type EGFR, results in the inhibition of mTOR which is a key intracellular kinase involved in the regulation of proliferation and cell survival [36,37]. The mTOR blockade, therefore, interferes at multiple levels with tumor growth, and the dual targeting of EGFR and mTOR may have a greater effect with respect to the administration of a single target [4]. However, STAT3, which is persistently activated in 22–65% of NSCLC [20,38], remains in an active state. Overall, the inhibition of the EGFR signaling pathway can be responsible for the inhibition of cell growth and apoptosis activation seen in the A549 cells. Cellular migration is reduced only at IC50 EGCG concentration or more (70 and 90 µM). Additionally, in this case, the decrease in mVnc could be involved in the observed decline in cell motility (Figure 5). The most EGCG-responsive NSCLC, in terms of both signaling pathway activation and cell migration, is, however, the HCC827 cell line in which all analyzed EGFR downstream pathways, from mTOR to MAPK38, are suppressed. Considering that the use of TKIs induces STAT3 and MAPK38 activation [39,40,41], the use of EGCG as an adjuvant drug can effectively increase the efficacy of TKIs in this specific type of NSCLC. The inhibition of these pathways affects cell proliferation, explaining the higher impact that EGCG treatment elicits in HCC827 cells. Moreover, in these NSCLC cells, migration is arrested at all EGCG concentrations tested and, only for this NSCLC cell line, a significant reduction in Vnc is observed, therefore lowering its invasive and metastatic potential.

## 4. Materials and Methods

### 4.1. Reagents

Cell culture reagents were obtained from Euroclone (Milan, Italy). Chemical reagents and propidium iodide (PI) were obtained from Sigma-Aldrich (St. Louis, MO, USA). Annexin V-FITC apoptosis detection kit was obtained from Biolegend (San Diego, CA, USA). Chemiluminescent substrate and secondary antibody (32460) were obtained from Thermo Fisher Scientific (Waltham, MA, USA). Bradford reagent and polyvinylidene difluoride (PVDF) membranes were obtained from Bio-Rad (Hercules, CA, USA).

### 4.2. Cell Culture

The human non-small cell lung cancer (NSCLC) cell lines were all obtained from the American Type Culture Collection (ATCC). The H1975 (CRL-5908™) and HCC827 (CRL-2868™) cell lines were grown in RPMI 1640 medium (ECB2000) supplemented with 10% fetal bovine serum (FBS), 2 mM L-glutamine, 100 U/mL penicillin, and 100 μg/mL streptomycin while the A549 (CCL-185™) was grown in complete DMEM/F12 medium (ECM0095). All cell lines were routinely maintained in 75 cm^2^ flasks at 37 °C, 5% CO_2_, and 95% relative humidity. The cell cultures were detached by trypsinization with 0.5% trypsin in PBS containing 0.025% EDTA and counted using trypan blue exclusion assay. All cell culture reagents were supplied by Euroclone. For all treatments, EGCG was freshly prepared in deionized water at a concentration of 12 mM.

### 4.3. Cell Viability Assay

The number of metabolically active cells, and thus cell viability, was assessed by a 3-(4,5-dimethylthiazol-2-yl)-2,5-diphenyltetrazolium bromide (MTT) assay [12,42]. The NSCLC cell lines (A549, H1975, and HCC827) were seeded in 24-well plates to reach 50% confluence at 24 h; then, the medium was removed and replaced with 1 mL of fresh culture medium containing increasing EGCG concentrations (0–640 µM). After 72 h, the medium from each well was removed and replaced with fresh medium supplemented with MTT at a final concentration of 100 µg/mL, and the NSCLC cells were incubated for 3 h at 37 °C in a 5% CO_2_ atmosphere. Then 0.4 mL of DMSO was added to each well to solubilize the purple formazan crystals formed from MTT reduction. The absorbance was read on a multiwell scanning microplate reader (BioTek Synergy HT MicroPlate Reader Spectrophotometer, BioTek Instruments Inc., Winooski, VT, USA) at 570 nm using the extraction buffer as a blank. The optical density in the control group (untreated cells) was considered as 100% viability. The relative cell viability (%) was calculated as (OD_570_ of treated samples/OD_570_ of untreated samples) × 100. Dose-dependent curves were therefore generated for the cytotoxic studies. The 50% inhibiting concentration (IC50) was determined by non-linear regression analysis with a three-parameter fit by utilizing SigmaPlot 12.0 Software. Each experiment was performed at least five times in triplicate.

### 4.4. EGFR Phosphorylation Studies by Flow Cytometry

The effect of EGCG on EGFR phosphorylation inhibition was evaluated by flow cytometry. For induction of EGFR phosphorylation, A549 cells were incubated for 20 min with 50 ng/mL recombinant EGF (Euroclone), before EGCG treatment. The NSCLC cell lines were treated with 30, 70, and 90 μM EGCG for 4 h. Cells were harvested by incubation with 0.25% trypsin-EDTA and fixed for 20 min at room temperature (R.T.) with 2% paraformaldehyde. Before the staining with antibodies against EGFR (Invitrogen, MA5-28104) and phospho-EGFR (Y1068) (R&D System, IC3570F), the cells were permeabilized for 30 min on ice in PBS, 0.5% BSA and 0.025% Triton X100. Anti-EGFR and anti-phospho-EGFR were incubated for 1 h in the dark at 4 °C. At least 5000 cells for each sample were measured using the same settings. For inhibiting potential phosphatase activity, the staining and permeabilization buffers were supplemented with sodium fluoride (NaF) and sodium orthovanadate (Na_3_VO_4_) to a final concentration of 1 mM. The percentage of EGFR phosphorylation was calculated by dividing the geometric mean fluorescence intensity of phospho-EGFR by the geometric mean fluorescence intensity of the EGFR.

### 4.5. Apoptosis Assay

Apoptosis was analyzed by flow cytometry using Annexin V-FITC apoptosis detection kit (Biolegend), according to the manufacturer’s instructions. A549, HCC827, and H1975 cells were treated with different EGCG concentrations (30, 70, and 90 µM). The analysis was performed after 72 h. Briefly, control and treated cells were trypsinized, washed twice with ice-cold PBS, and resuspended in 1X Annexin binding buffer at a final concentration of 1.0 × 10^6^ cell/mL. Annexin V-FITC (0.25 µg/mL) and PI (1 µg/mL) were added to the cell suspension and the mixture was incubated for 15 min at R.T. in the dark [35]. Samples were analyzed using the Guava EasyCyte flow cytometer (Millipore) at an excitation wavelength of 488 nm. A total of 5000 events were acquired for each sample. Annexin V-FITC was detected as a green fluorescence and PI was detected as a red fluorescence. Early apoptosis is defined by Annexin V+/PI− staining, late apoptosis is defined by Annexin V+/PI+ staining, and necrosis is defined by Annexin V−/PI+ staining.

### 4.6. Cell Migration Analyses

A549, H1975, and HCC827 cell migration were evaluated by the scratch wound assay. Briefly, the cell lines were seeded on 6-well plates. After 24 h, scratch wounds were created with 1000 μL pipette tips on the pre-seeded confluent cells. After scratch wound induction, the culture was washed with PBS and replaced by a fresh medium containing increasing concentrations of EGCG (30, 70, and 90 µM). Photomicrographs were taken at time 0 (immediately following the scratch wound), 24, 48, and 72 h. The wound gaps were measured by ImageJ (version 1.47; NIH, Bethesda, MD, USA). The percentage migration was calculated by the average area reduction at 24, 48, and 72 h as compared to time 0. Every well had 5 scratch wounds.

### 4.7. Western Blot

Cells were lysed with RIPA buffer (50 mM Tris-HCl pH 8.0, 150 mM NaCl, 2 mM EDTA, 0.5% Triton X-100) containing protease inhibitor cocktail (Sigma-Aldrich, P8340) on ice for 40 min and centrifugated at 12,000× *g* for 10 min at 4 °C. Supernatants were collected and protein concentration was determined by the Bradford assay. Equal amounts of denatured lysates (20 μg) were separated on 4–20% precast SDS-PAGE system (Bio-Rad) and then transferred onto immunoblot PVDF membrane. The membrane was blocked with EveryBlot blocking buffer (Bio-Rad) for 5 min at 25 °C. After blotting, the membrane was cropped at the height of the protein of interest before being incubated with the specific antibody. Several antibodies can be visualized in the same run and the GAPDH is the same for all. The cropped blots were incubated with rabbit monoclonal antibodies (Cell Signaling Technology) against mTOR (#2983), phospho-mTOR (Ser2448) (#55336), STAT3 (#4904), phospho-STAT3 (Tyr705) (#9145), p38 (#8690), and phospho-p38 MAPK (Thr180/Tyr182) (#9211) at 4 °C overnight. All antibodies were diluted at a 1:2000 ratio. Mouse monoclonal anti-vinculin (1:300, Invitrogen, MA5-11690) was incubated for 1 h at R.T. All membranes were washed three times with TBST for 10 min and incubated with specific horseradish peroxidase (HRP)-conjugated secondary antibodies in accordance with the manufacturer’s instructions for 1 h at R.T. Membranes were developed with enhanced SuperSignal™ west pico plus chemiluminescent substrate (Thermo Fisher Scientific) and optical densities were analyzed by ChemiDoc™ gel imaging system (Bio-Rad) and analyzed using ImageJ (version 1.47; NIH, Bethesda, MD). As an internal control for protein loading, membranes were reprobed with rabbit monoclonal GAPDH antibody (Cell Signaling Technology #2118) for 1 h.

### 4.8. Statistical Analyses

Data are presented as mean ± S.D. (standard deviation). Statistical comparison of differences among groups of data was carried out using one-way analysis of variance (ANOVA), followed by Tukey’s post hoc test using GraphPad Prism. Values of *p* < 0.05 were considered statistically significant and values of *p* < 0.01 were considered highly significant.

## 5. Conclusions

The effect of EGCG is NSCLC type-specific. The EGFR signaling pathway inhibition seems to be directly correlated to the ability of EGCG to influence the phosphorylation status of EGFR. In fact, in the H1975 cell line, in which EGCG is not able to inhibit EGFR phosphorylation, no changes are observed in the phosphorylation status of mTOR, MAPK38, and STAT3. In the other two cell lines the EGFR inhibition results instead in the alteration of the phosphorylation of these proteins. However, further studies need to be performed to assess whether EGCG directly inhibits the EGFR-mediated signaling pathway. The cells in which this inhibition occurs (A549 and HCC827) appear to be the most EGCG-sensitive NSCLC cells. In addition, EGCG also induces apoptosis in H1975 cells indicating that catechin acts by modulation of other pathways. In all NSCLC analyzed, EGCG treatment reduces cell migration and induces changes in vinculin and meta-vinculin expression. Overall, this makes the EGCG a potential adjuvant drug for lung cancer therapy.

## Data Availability

Not applicable.

## Supplementary Materials

Multiple exposures of Western blot analysis are available online at https://www.mdpi.com/article/10.3390/ijms222111833/s1.
