# Supplementary material for: Effect of Epigallocatechin-3-Gallate on EGFR Signaling and Migration in Non-Small Cell Lung Cancer
Source: Int J Mol Sci. 2021 Oct 31;22(21):11833. doi: 10.3390/ijms222111833 (PMC8583909; doi:10.3390/ijms222111833)
Supplement: Supplementary file 1 [file ijms-22-11833-s001.zip › ijms-1396736-supplementary.pdf]

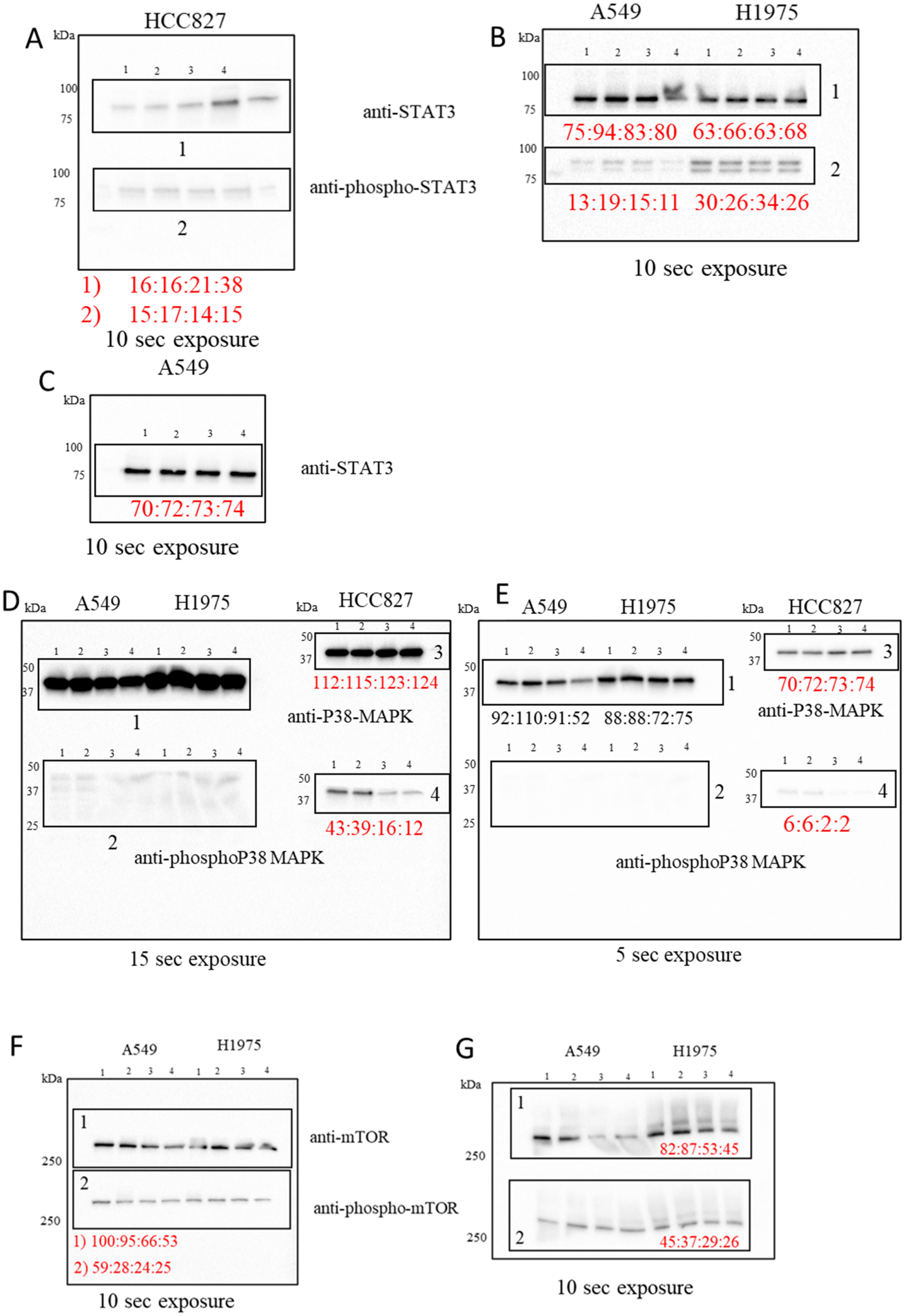
A
HCC827
kDa
1 2 3 4
100
75
1
anti-STAT3
100
75
2
anti-phospho-STAT3
1) 16:16:21:38
2) 15:17:14:15
10 sec exposure
B
A549
H1975
kDa
1 2 3 4 1 2 3 4
100
75
1
75:94:83:80 63:66:63:68
100
75
2
13:19:15:11 30:26:34:26
10 sec exposure
C
A549
kDa
1 2 3 4
100
75
anti-STAT3
70:72:73:74
10 sec exposure
D
kDa
A549
H1975
kDa
HCC827
1 2 3 4 1 2 3 4
50
37
1
1 2 3 4
50
37
3
112:115:123:124
anti-P38-MAPK
1 2 3 4 1 2 3 4
50
37
25
2
anti-phosphoP38 MAPK
1 2 3 4
50
37
4
43:39:16:12
15 sec exposure
E
kDa
A549
H1975
kDa
HCC827
1 2 3 4 1 2 3 4
50
37
1
92:110:91:52 88:88:72:75
1 2 3 4
50
37
3
70:72:73:74
anti-P38-MAPK
1 2 3 4 1 2 3 4
50
37
25
2
1 2 3 4
50
37
4
6:6:2:2
anti-phosphoP38 MAPK
5 sec exposure
F
A549
H1975
1 2 3 4 1 2 3 4
kDa
1
250
anti-mTOR
2
250
anti-phospho-mTOR
1) 100:95:66:53
2) 59:28:24:25
10 sec exposure
G
A549
H1975
1 2 3 4 1 2 3 4
kDa
1
250
82:87:53:45
250
2
45:37:29:26
10 sec exposure

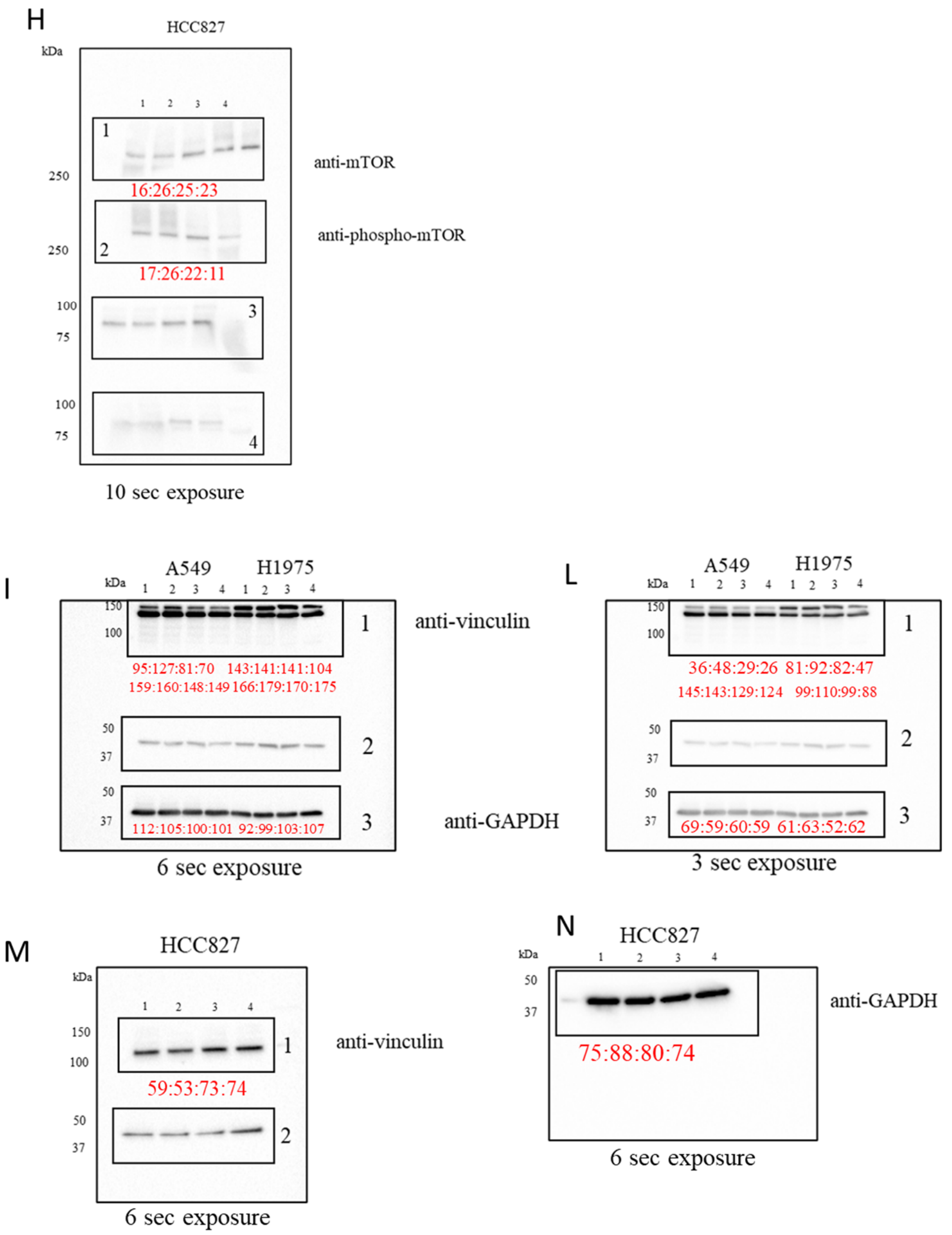

Supplementary Figure S1. ***Multiple exposures of western blot analysis***. The number of samples did not allow for a single run. The sample derive from the same experiment, gels and blots were processed in parallel. After blotting, membranes were cropped, with the help of the marker, at the height of the protein of interest before being incubated with the antibody. In this way, several antibodies with different incubation modes can be visualized in the same run. During development at ChemiDoc, the cropped membranes were developed together considering the sensitivity of the antibody and the same exposure times. In the figure, letters A–N show the ChemiDoc images, while the numbers the different membranes. The following images were used: C (A549), A1 (HCC827) and B1 (H1975) were used for anti-STAT3 antibody and AE2 and B2 for phosho-STAT3. For p38-MAPK D3 and E1 were used, while D4 and E2 were used for phospho-p38-MAPK membranes. Figure F (A549) and G-H (H1975 and HCC827) were used for anti-mTOR and anti-phospho-mTOR membranes. Figure L1 and M1 were used for anti-vinculin antibody, and I3 and N for GAPDH antibody. Sample legend: 1-untreated; 2- EGCG, 30 μM; 3- EGCG, 70 μM; 4- EGCG, 90 μM.
